# Supplementary material for: Reconfigurable magnonic mode-hybridisation and spectral control in a bicomponent artificial spin ice
Source: Nat Commun. 2021 May 3;12:2488. doi: 10.1038/s41467-021-22723-x (PMC8093262; doi:10.1038/s41467-021-22723-x)
Supplement: Supplementary file 1 — Supplementary Information [file 41467_2021_22723_MOESM1_ESM.pdf]

Reconfigurable magnonic mode-hybridisation and spectral control in a bicomponent artificial spin ice

Jack C. Gartside[1,3,*], Alex Vanstone[1,3], Troy Dion[1,2], Kilian D. Stenning[1], Daan M. Arroo[2], Hide Kurebayashi[2], and Will R. Branford[1]

[1]Blackett Laboratory, Imperial College London, London SW7 2AZ, United Kingdom
[2]London Centre for Nanotechnology, University College London, London WC1H 0AH, United Kingdom
[3]These authors contributed equally to this work
[*]Corresponding author e-mail: j.carter-gartside13@imperial.ac.uk

Supplementary Information

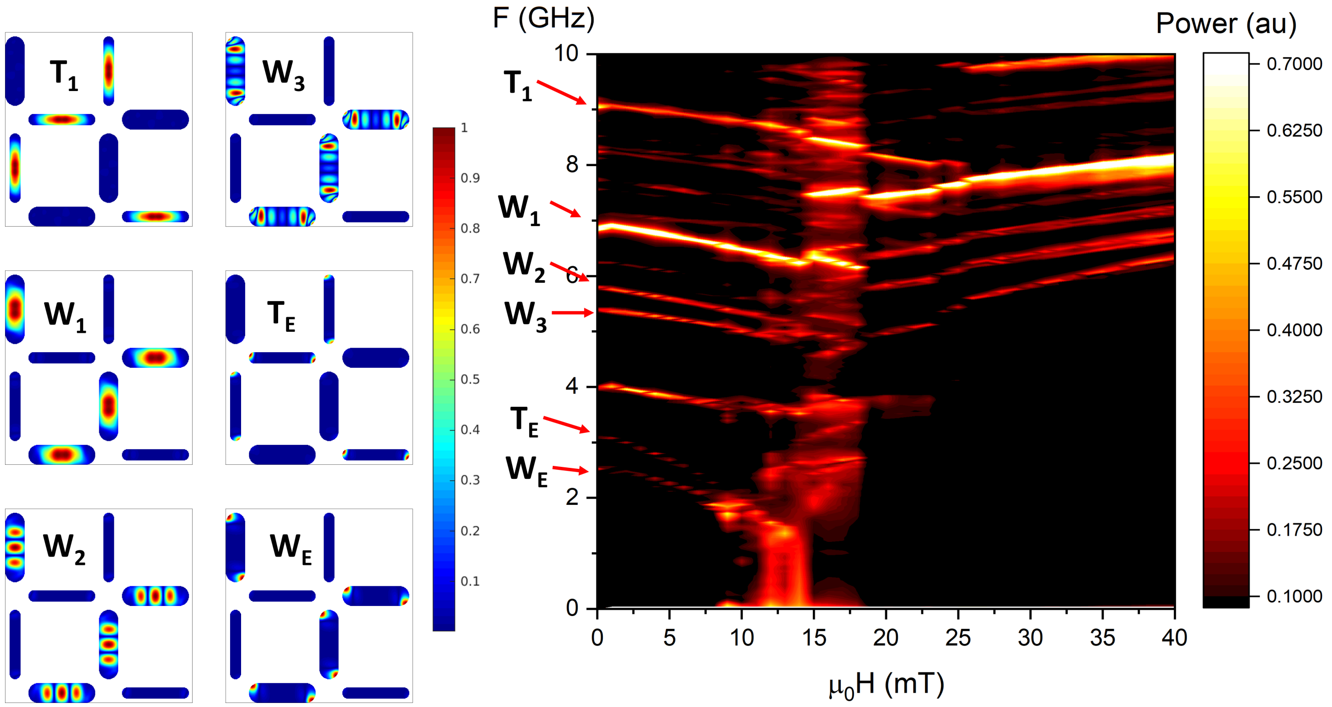

Supplementary figure 1. Simulated spatial mode profiles of the S sample ('monopole' orientation) taken at zero field along with corresponding 0-40 mT spectra.

Supplementary note 1 - Simulated spatial mode profiles

Micromagnetic simulations of spatial mode profiles taken at zero field and corresponding 0-40 mT spectra for the S sample ('monopole' orientation) are shown in supplementary figure 1. Mode labelling numbers W1, W2 and W3 are sequential and do not denote mode index number.

Supplementary note 2 - Extended frequency and field range spectra of type 3 spectra

Supplementary figure 2 shows simulated spectra for the HDS sample ('monopole' orientation) prepared in the type 3 state. Corresponding to an wider frequency range version of the spectra shown in figure 3 e), the edge modes of the thin and wide bars are observed in the 0-3 GHz frequency range. The wide bar edge mode reverses gradient at -1.5 mT, the thin bar edge

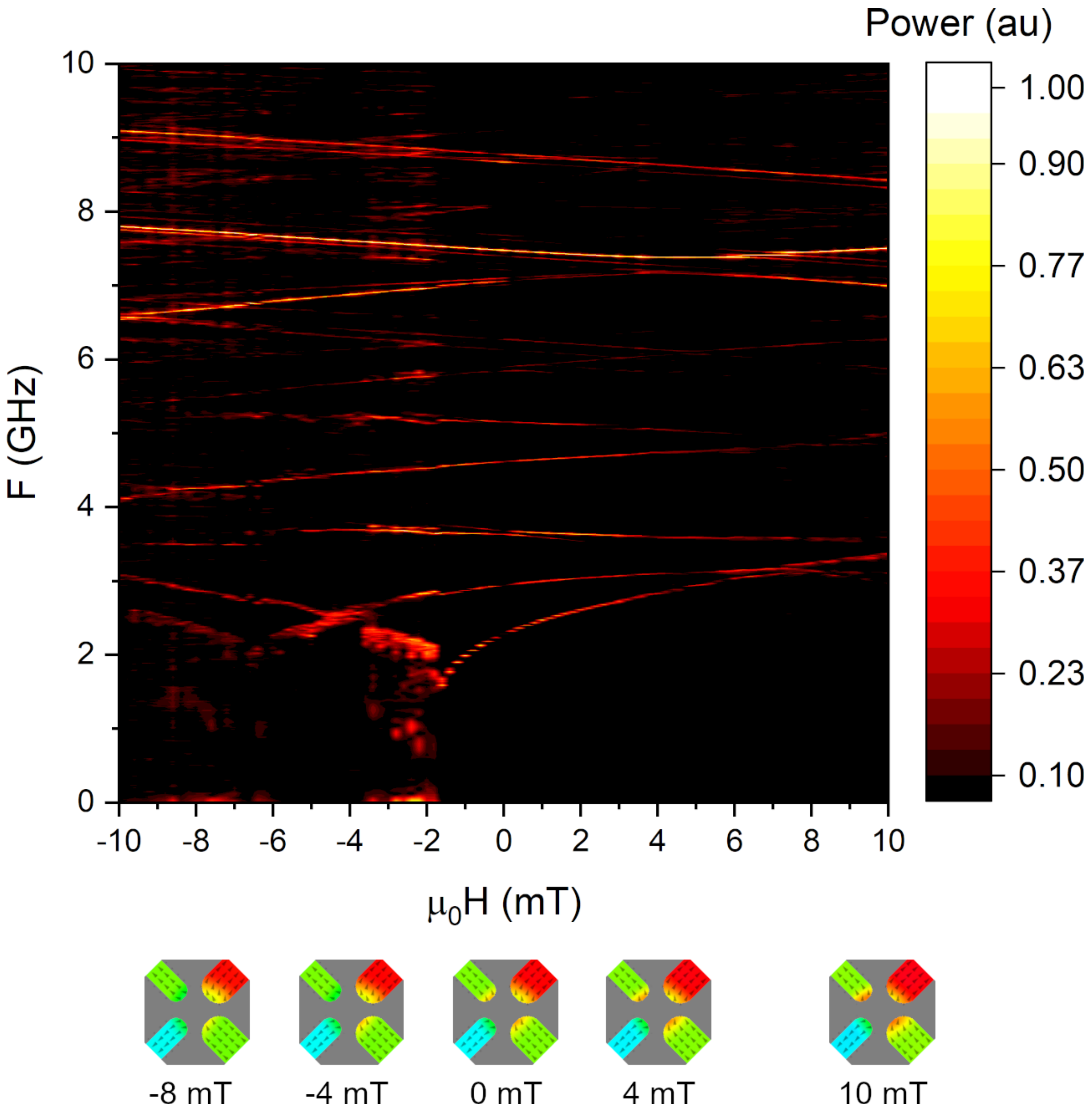

Supplementary figure 2. Simulated spectra of HDS sample ('monopole' orientation) prepared in type 3 microstate. 0-10 GHz frequency range shows edge mode behaviour (0-3 GHz range) of wide and thin bars and anticrossing of wide bar bulk modes at 5 mT, 7.2 GHz.

mode reverses gradient at -6.5 mT. These gradient reversals are caused by changes in the static magnetisation curl states of the nanoisland edge regions, which are shown at -8, -4, 0, 4 and 10 mT below the spectra. These state changes occur far from the anticrossing point at 5 mT and as such, rearrangements of the static edge magnetisation states are unlikely to be linked to the mode hybridisation behaviour.

Supplementary note 3 - Positions of the MOKE magnetization plateaux

Bar reversal occurs by a domain wall nucleation process at a field determined by the aspect ratios of the bars. The wide bar subset can be characterized by a mean coercive field H_{c1}, and a magnetization M_{wide} and the thin bar subset by a mean coercive field H_{c2} and a magnetization M_{thin}. Because the other bar dimensions are identical the ratio of the volumes and magnetisations is the same as the ratio of the bar widths. For a sample with all bars identical, the type 1 and type 4 state should have zero net magnetization. For the width modified sample the different volumes of reversed and unreversed bars would be expected (for perfect Ising spins and nominal bar widths) to give $M/M_{type2} = \frac{M_{wide}-M_{thin}}{M_{wide}+M_{thin}} = \frac{t_{wide}-t_{thin}}{t_{wide}+t_{thin}}$ = 0.226 for the S sample and 0.231 for the HDS sample (for both type 1 and type 4). The relative magnetization of the ground-state minor loop to the saturated major loop at zero field is approx. 0.3 in the S sample and 0.2 in the HDS sample. For the type 4 state it is approx. 0.5 and 0.6 for S and HDS respectively. In a type 3 that is formed by both wide bars switching and triggering one thin bar to also reverse, then M/M_S would be $\frac{t_{wide}}{t_{wide}+t_{thin}}$ = 0.613 for S and 0.615 for HDS. Imperfections in the nanofabrication (quenched disorder) give the sublattice switching fields a Gaussian distribution about the mean, with a standard deviations σ_{wide} around H_{c1} and σ_{thin} around H_{c2}. If bars were all sufficiently spaced to be not interacting then desired states could be accessed by applying $H_{ext} = H_{c1} + H_{c2}/2$ as long as $H_{c2} - H_{c1} >> \sigma_{wide} + \sigma_{thin}$. However we are in the strongly-interacting regime and so each bar experiences an effective field $H_{eff} = H_{app} + H_{loc}$. The reversal of wide bars will change H_{loc} experienced by the thin bars. If ΔH_{loc} increases H_{eff} then this makes it more difficult to realise the ordered state, as do the cases where we are preparing a type 4 state. Where we are writing the type 1 state from the saturated type 2 state, ΔH_{loc} decreases H_{eff} and so the interactions increase the operating window where the ordered state may be prepared. The difference can be seen in the MOKE hysteresis loops in fig. 1 c,d,e,f). In fig. 1 c,e) we have very clear plateaux in the major (blue) hysteresis loops and can very easily and reproducibly send minor loops to the type 1 microstate and back to saturated. Note that the data is the average of thousands of individual loops and so the sharp switching and flat plateuax show there is no significant stochasticity in this major hysteresis loop and we go through the same microstates at the same fields in each measurement. Similarly in the minor (orange) loops we repeatedly go the the same expected plateau magnetization. For the same sample, with the same extrinsic disorder and sigmas, in the monopole geometry, switching the wide bars causes the dipolar field of all neighbouring bars to help the reversal of the thin bars and so the two Gaussian distributions start to overlap. We know from MFM we can access large areas of pure type 4 with the correct protocol, but the hysteresis loop shows very broad reversal with no clear plateaux. It is not clear from our data whether the broadening we see is from averaging similar broad loops or different loops with sharper individual features. The disorder could be spatial within the measurement spot, temporal with loop cycle number or both. Certainly there is a significant stochastic contribution in the measurement.
